# A Case Report of Mesothelioma Response to Endocrine Therapy in Synchronous Breast Cancer and Pleural Epithelioid Mesothelioma: A Double Exemestane Effect

**DOI:** 10.7759/cureus.31579

**Published:** 2022-11-16

**Authors:** Marta Pina, Rute Fernandes, Diogo Fonseca, Cristina Oliveira, Ana Rodrigues

**Affiliations:** 1 Medical Oncology, Instituto Português de Oncologia do Porto, Porto, PRT; 2 Radiology, Instituto Português de Oncologia do Porto, Porto, PRT

**Keywords:** exemestane, endocrine therapy, synchronous tumours, breast cancer, pleural mesothelioma

## Abstract

Synchronous pleural mesothelioma (PM) and breast cancer are extremely rare. We present the case of a 53-year-old female diagnosed with localized breast cancer. She was radically treated with surgery, but during the adjuvant radiotherapy, the patient developed fever and dyspnoea, and pleural thickening was found on a CT scan. The biopsy confirmed the diagnosis of a synchronous malignancy - pleural epithelioid mesothelioma.

The patient stopped radiotherapy and started adjuvant endocrine therapy with exemestane, a third-generation aromatase inhibitor, with an unexpected partial response to the PM. The patient remains on exemestane with a sustained partial response.

This is a rare case of synchronous tumours that show a real-life benefit of exemestane in the treatment of pleural mesothelioma, which was only described in vitro, with a good sustained response. This suggests a potential for exemestane in the treatment of mesothelioma, which is an aggressive form of cancer with few therapeutics with sustained results.

## Introduction

Malignant mesothelioma (MM) is an aggressive form of cancer that arises from mesothelial surfaces in the pleura, peritoneum, pericardium, or tunica vaginalis testis. There are three histological subtypes: epithelial, sarcomatoid, and biphasic. This classification has diagnosis and prognosis implications, but not for treatment [1,2].

Pleural mesothelioma (PM) is the most common form and accounts for 65-70% of all cases of mesothelioma. Diffuse dissemination throughout the pleural cavity is common at presentation and is frequently associated with symptomatic and massive pleural effusion, with an impact on the quality of life of patients [1,2].

The risk of disease increases with age and is higher in men. In previous studies, female patients have been reported to have better overall survival than male patients (17.3 months compared to 11.8 months, respectively) [1].

There is a well-established causal relationship between asbestos exposure and MM, with this association being particularly strong in PM, where 80% of patients report a history of asbestos exposure, even though the latency period can be long [2].

The standard therapeutic modalities for PM might include surgery, chemotherapy (cisplatin 75 mg/m^2^ + pemetrexed 500 mg/m^2^ every 21 days with or without immunotherapy), and radiotherapy, yet with unsatisfactory outcomes. In general, even with adequate treatment, mesothelioma has a very poor prognosis, with a median survival from the time of presentation of approximately 9-18 months [1-4]. Therefore, in order to improve the clinical outcome of the pharmacological treatment of this refractory tumour, drugs that target novel and/or specific tumoral targets are needed.

Only a few cases of synchronous tumours with PM have been described in the literature, and usually they are both derived from asbestos exposure, like primary bronchogenic neoplasms. However, here are some reports on metachronous mesothelioma following radiotherapy for the treatment of primary breast cancer. Breast cancer is the most common cancer among women and can be associated with other primary cancers via germline mutations, including BRCA1/BRCA2, and p53 [4-7].

To the best of our knowledge, this is the first published case of pleural malignant mesothelioma with synchronous breast cancer.

## Case presentation

We presented the case of a 53-year-old woman with Eastern Cooperative Oncology Group performance status (ECOG-PS) 0, meaning fully active and able to carry on all pre-disease functions without restriction. She has no relevant past medical history. She was a non-smoker without known exposure to asbestos and used oral contraceptives for five years.

**Figure 1:**
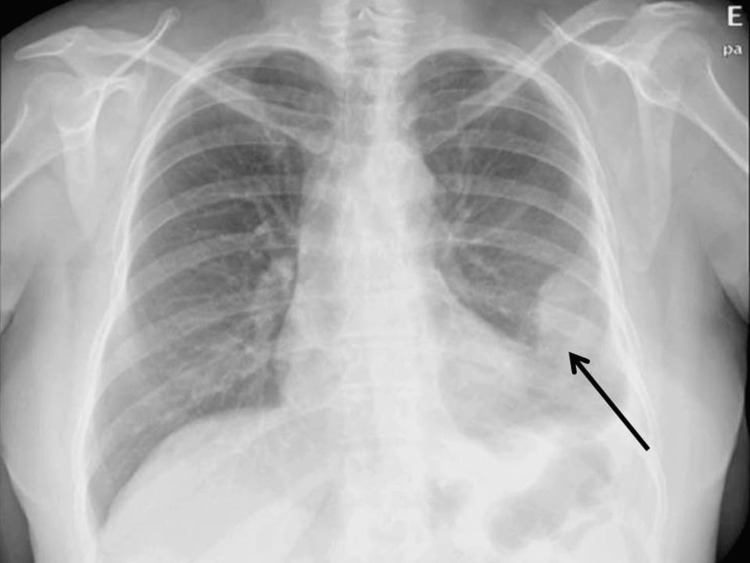
Chest X-ray - May 2021, with hypodensity at left lung base (arrow)

The patient was asymptomatic when, in breast screening tests, a suspicious lesion was detected in November 2020. In March 2021, she was scheduled for a partial left mastectomy with a sentinel ganglion biopsy. The histology confirmed ductal invasive carcinoma, luminal A, stage IA (pT1bN0sn cM0). The patient was proposed for adjuvant treatment with radiotherapy and endocrine therapy. Exemestane 25 mg/day (a third-generation aromatase inhibitor) was started in April 2021. In May 2021, she started radiotherapy on the left breast. On the sixth day of radiotherapy, the patient developed a febrile syndrome and malaise. A chest X-ray was performed and showed a hypodensity at the left lung base (Figure 1).

**Figure 2:**
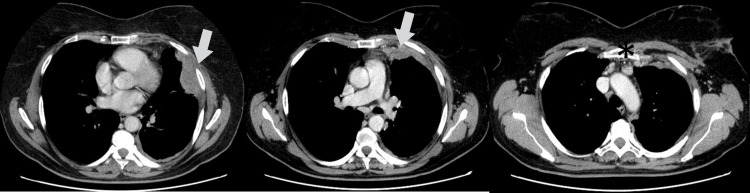
Computed tomography scan (May 2021) - pleural thickness (arrows) and 15 mm adenomegaly (*)

To complement the study, a CT scan was performed, and it showed a small left pleural effusion, pleural thickening with multiple implants, the largest with 53 mm × 25 mm, and suspicious lymph nodes in the internal mammary chain and prevascular mediastinal lymph node, the largest with a short axis of 13 mm (Figure 2).

The PET-CT confirmed the presence of metabolic activity extensively involving the left pleural surface, unspecific small lymph nodes in the left axilla, and alterations in the left breast compatible with the sequelae of the surgical procedure. However, these findings were decreasing in size when compared to the CT scan performed 30 days earlier.

The histologic result of the pleural biopsy was compatible with malignant epithelioid mesothelioma (positivity for CK5/6, CK7, WT-1, and D2-40 and negative for GATA3, TTF-1, PAX-8, and hormonal receptors).

After a discussion with a breast cancer multidisciplinary team, taking into consideration the diagnosis of a second malignancy, the adjuvant radiotherapy was suspended after seven days of treatment. Hormonotherapy with exemestane was maintained.

**Figure 3:**
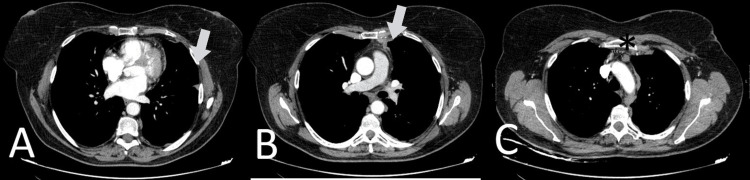
Computed tomography scan (June 2022) - with significant reduction of pleural thickness (arrows), and 11 mm adenomegaly (*)

The patient's case was discussed with a thoracic tumour multidisciplinary team, and the mesothelioma was considered not suitable for local resection or chemoradiation. Nevertheless, considering the partial response assumed in relation to the ongoing hormonotherapy with exemestane, the patient was proposed to maintain the treatment with close follow-up. In July 2022, the patient was clinically asymptomatic, still on exemestane with very good tolerance, and the CT scan showed a sustained partial response (Figure 3).

## Discussion

A synchronous tumour is defined as a second tumour diagnosed within six months of the primary tumour. Synchronous cancers provide a therapeutic challenge because tumour stage, behaviour, and prognosis may be different from individual malignancies [8].

There are several cancer predisposition syndromes that increase the risk of synchronous and metachronous cancers, like Li Fraumeni and Beckwith-Wiedemann syndromes, Cowden syndrome, and BRCA gene mutations, etc.; exposure to the same risk factors (such as asbestos, tobacco, alcohol, late effects of chemotherapy or radiotherapy, etc.); advanced age; and improved screening and diagnostic exams also contribute to increased diagnosis [9].

Due to the rarity of these tumours, there is still no consensus on the definitive treatment protocols. As a result, individualized treatment with multidisciplinary close follow-up might improve survival outcomes [4-7].

Given that women have a lower risk of developing mesothelioma and have a higher survival rate when compared to men, some studies hypothesized that hormones, in addition to epidemiological differences such as occupational trends, could be a factor [10].

Some recent studies have shown the role of aromatase (CYP19A1) in patients with PM. CYP19A1 is a key enzyme in the biosynthesis of oestrogen (converting testosterone into estradiol). Exemestane is an inhibitor of CYP19A1 type 1 (a steroidal inactivator), which induces cell death in Ist Mes1, Ist Mes2, and MPP89 MP cell lines, and there are studies that suggest it can be effective alone and in combination with chemotherapy [1,11-13].

These preclinical data support the clinical benefit seen in this patient, as our patient was taking exemestane as adjuvant hormonotherapy for breast cancer, but we had a clinical benefit and an objective radiological response in the PM.

## Conclusions

Synchronous tumours are rare, and, to the best of our knowledge, this is the first clinical case of a patient with synchronous breast cancer and pleural mesothelioma. The fact that this patient was first being treated for breast cancer gave us the chance to realize the effect of exemestane in vivo in a PM, supporting the results of the preclinical data already published. Also, it may provide a treatment option with better tolerability than chemotherapy for aggressive and highly symptomatic diseases after failure with standard treatments.

This patient has been on exemestane for 14 months with good response, tolerance, and quality of life. This gives us hope for the patient's long survival, but also for a therapeutic opportunity to explore other patients.
